# Benefits of Magnesium Supplementation to Broiler Subjected to Dietary and Heat Stress: Improved Redox Status, Breast Quality and Decreased Myopathy Incidence

**DOI:** 10.3390/antiox8100456

**Published:** 2019-10-07

**Authors:** Mario Estevez, Massimiliano Petracci

**Affiliations:** 1 IPROCAR Research Institute, Food Technology, University of Extremadura, 10003 Cáceres, Spain; 2 Department of Agricultural and Food Sciences, Alma Mater Studiorum-University of Bologna, 47521 Cesena, Italy; m.petracci@unibo.it

**Keywords:** oxidative stress, poultry, magnesium, meat quality, myopathy

## Abstract

Poultry is highly sensitive to oxidative reactions. Oxidative reactions have attracted considerable attention from animal and food scientists because of the adverse effects of these reactions on animal welfare, performance and food quality. Despite its implication in multiple biological functions magnesium (Mg) supplementation is typically overlooked in broiler diets. The objective of this study was to evaluate the effect of Mg supplementation (0.3%) using a commercial product (Optibreast^®^) on production parameters, the redox status and meat quality in broilers challenged with dietary (oxidized oil) and heat stress. The incidence of myopathies, namely, wooden breast (WB) and white striping (WS) was also assessed. Mg supplementation had a clear interaction with the absorption/accumulation of Ca in blood and breast muscle but this effect had no negative influence on any of the production parameters under study. Mg supplementation had positive effects on particular meat quality traits such as water holding capacity (WHC) and color. WHC may have other positive effects in turn on relevant sensory traits such as juiciness. Mg supplementation protected against protein oxidation in liver and plasma of broilers. This effect may be related to the increased activity of catalase in such tissues. Mg supplementation reduced the incidence of WS and WB myopathies to almost half the occurrence of such defects in animals fed a control diet. Further studies with a larger number of animals and the application of advanced proteomic/metabolomic tools are required to (1) corroborate the positive influence of Mg on myopathy incidence and (2) identify the underlying molecular basis of the proposed mechanisms.

## 1. Introduction

Feeding accounts for the largest percentage of the total cost in the poultry industry, and the influence of the dietary background on production parameters and poultry meat quality is indisputable. The optimization of the feeding ingredients and supplements is essential to adjust production costs and, additionally, guarantee consumer’s acceptance of the final product [1]. The impact of feeding on meat quality has been extensively documented and it is generally known that feed management, quantity and composition affect the physicochemical properties and overall sensory quality of chicken meat [2]. This is of the utmost practical relevance as the main purpose of poultry production is to keep the final consumer satisfied, therefore feeding composition and management should be considered with care to guarantee satisfactory levels of sensory and nutritional quality in the chicken meat. Oxidative stress is a major threat to poultry production and meat quality, and it is known that dietary management can actually improve the oxidative stability of animal tissues and poultry meat [2,3]. Oxidative stress is the result of the imbalance between pro-oxidants and the endogenous antioxidant defenses and leads to uncontrolled production of reactive oxygen species (ROS) and oxidative damage to cellular components [3]. Among animal-source foods, meat from domestic birds is known to be particularly susceptible to oxidative reactions due to the high concentration of polyunsaturated fatty acids [4]. However, the susceptibility of muscle tissue to suffer oxidative processes is also driven by many other factors including endogenous (i.e., myoglobin concentration, occurrence of antioxidant enzymes) and external factors. Among the latter, pre-slaughter stress, postmortem changes (pH decline, oxygen exposure, etc.), processing (high temperatures, salting, curing, size-reduction processes, etc.) and storage conditions (length, temperature, and packaging) are among the most remarkable [5]. In the living birds, oxidative stress is a relevant source of biological damage, occurrence of diseases and impaired poultry growth [6]. The onset of rancidity and off-flavors in processed and subsequently stored poultry products is identified as one of the most salient drawbacks of lipid oxidation (LOX) [5]. In order to avoid these undesirable effects of LOX, assorted antioxidant solutions against poultry meat oxidation are commonly applied, and involve both nutritional (feed-based) and technological (formulation/packaging) strategies [2]. For the former, the application of phytochemicals and other specific micronutrients are of increasing interest [6]. Among the latter, selenium (both organic and inorganic) has been a focus of scientists owing to its involvement in the strengthening of the endogenous antioxidant defenses. Numerous studies have highlighted the role of this element in production and reproduction parameters, poultry meat quality and preservation against oxidative reactions [7]. Many other micronutrients are known to be essential for assorted biological functions but their role in the endogenous antioxidant defenses and the overall oxidative stability of poultry tissues is not so well understood. Magnesium is a good example of an essential element with recognized biological functions (i.e., muscle and bone growth) and limited knowledge about its influence on the oxidative stability and overall quality of poultry meat. 

Additionally, the poultry industry faces nowadays a major challenge with emerging myopathies such as the white striping and the wooden breast, which are believed to be related to fast growth [8]. Selected genetic lines and improved feeding programs have increased the growth rate, shortening the production cycle of broilers in the last few decades. However, this improvement may have been made at the expense of poultry meat quality. The link between the occurrence of these chicken breast defects and oxidative stress is not well defined but recent studies have shown higher oxidation rates in animals with these myopathies [9,10]. It is reasonable to hypothesize that animals with a higher growth rate and accelerated metabolism are more susceptible to oxidative stress owing to a more intense production of ROS. It is, once again, unknown to what extent dietary management could reduce the incidence of these unpleasant and very common chicken breast defects. 

According to the aforementioned, the objective of this study was to assess the effect of supplementation with a magnesium product (Optibreast^®^, Timab Magnesium, Dinard cedex, France) on redox status and breast meat quality of 42-day broilers submitted to a pre-slaughter oxidative stress. 

## 2. Material and Methods

### 2.1. Experimental Setting and Analyses at the Farm

This study was carried out in compliance with the Guidelines on Good Clinical Practice for Clinical Trials for Registration of Veterinary Medicinal Products. The animals used in the present scientific study were handled according to Regulation 2010/63/EU and following the recommendations of the European Commission 2007/526/CE. The study was approved by the pertinent Ethical Committee for Animal Research (file code: ABI+D2450616).

A total of 360 male and female Ross 308 broilers (sex ratio 1:1) from 1 to 42 days of age were used and allocated randomly to the experimental treatments. Before the trial, the animals were examined by a veterinarian for the occurrence of diseases, lesions, or any other health issue. Individuals showing any trace of a health disorder were discarded. Birds were observed on a daily basis in the search of appearance or behavioral anomalies. Casualties and slaughtered animals were registered for their weight, date and likely cause of death. The experiment was carried out in 18 pens (replicates) of an experimental farm with the stocking density being set at 10 broilers/m^2^. The experimental design was completely randomized with two dietary treatments (Table 1). Each treatment was replicated 9 times and 20 broilers housed together formed the experimental unit (*n* = 180 for each dietary treatment). The basal diet was based on wheat, corn and soybean meal with oxidized fat (10.95 meq active O_2_/kg of oil) as a source of dietary oxidative stress. A supplemented diet (Mg diet) was elaborated by including 3000 mg/Kg of a Mg commercial product (Optibreast^®^) provided by TIMAB Magnesium (Roullier group), to the basal diet. The Mg diet was supplied to the experimental broilers during the ‘growing’ (14–28 days) and ‘finishing’ periods (28–42 days). This dose was hypothesized to exert benefits in accordance to previous experiments and the scientific literature [7,11]. Feeds were made 2 weeks before the trial start to have time to check homogeneity (dry matter, ash, crude protein, ether extract, crude fiber and starch) and additives before trial start. Table 1 shows the full composition of experimental diets that were calculated according to the Spanish Foundation for the Animal Nutrition Development (FEDNA) tables [12]. The diets (mash) and water were provided ad libitum. The composition of and the analysis of the proximate composition (Table 1) was made following the Association of Official Agricultural Chemists (AOAC) methods for moisture (AOAC 930.15), ashes (AOAC 942.05), total protein (AOAC 984.13), total fiber (962.09; AOAC, 2007) and ether extract (AOAC 920.39). Magnesium was analyzed in experimental diets by ionic chromatography. Mg in the diets reflected the expected concentration of the aforementioned elements with less than 10% of variation from the formulated (in Table 1). The concentration of Mg in basal diet (no added TIMAB Mg product) was below 0.1%. 

The industrial housing unit was equipped with artificial illumination (18 h light and 6 of darkness), heating and a forced ventilation system. The temperature was set at 33–35 °C at the beginning of the trial and decreased 3 °C per week to stabilize at 24 °C for the 21 days of life. In the 48 h prior to slaughter, a heat stress was applied to all animals, increasing the temperature up to 30 °C during 8 h/day in 3–4 h intervals. The light program consisted in 18 h of light and 6 of darkness. Weight gains, feed intakes and feed conversion ratios of each pen were determined at 0, 14, 28 and 42 days of age, and calculated for each feeding period and for the whole experiment.

### 2.2. Sampling and Assessment of Carcasses for Myopathies

At the end of the experiment (day 42), 32 chickens per treatment (64 broilers in total) were randomly selected among those having homogeneous weights at slaughter (~2.4 ± 0.2 kg), euthanized by cervical translocation and immediately processed for sampling as explained below. Blood samples were collected from the heart of animals right after slaughter using two vacutainer needles with EDTA and heparin as clotting preventers. Subsequently, liver and breast muscles were carefully dissected and removed from carcasses. Chicken breasts were cleaned and immediately transferred to assessment of myopathies in accordance to a 3-point visual descriptive scale for WB (WB#1: normal, WB#2: slight bulging and toughness, and WB#3: moderate to severe bulging and toughness); and WS (WS#1: normal, WS#2: thin stripes < 1 mm, and WS#3: thick stripes > 1 mm). An assessment by consensus was carried out by three pathologists with more than 5 years of experience in the diagnosis of such myopathies following recommendations proposed by Petracci et al. [8].

### 2.3. Analysis of Biological Samples

#### 2.3.1. Minerals

Analyses of Mg^2+^ and Ca^2+^ in livers, muscles and blood were made by mineralization, subsequent reconstitution in acid environment and eventually identification and quantification by ion chromatography. Analysis of P in livers, muscles and blood was made by microwave-assisted digestion of sample, with subsequent analysis by inductively coupled plasma mass spectrometry (ICP-MS). The inter- and intra -assay coefficients of variation (CVs) were 12% and 6%, respectively. 

#### 2.3.2. Oxidation Markers

Analysis of MDA in livers, muscles and blood was made by an acidic extraction followed by reaction with thiobarbituric acid and subsequent reading of absorbance at 532 nm in accordance with the method proposed by Ganhão et al. [13]. Quantification was made using TEP (MDA analog) as standard. The inter- and intra -assay coefficients of variation (CVs) were 8% and 3%, respectively. 

Protein carbonyls were quantified using DNPH as derivatization agent and using spectrophotometry measurements at 370 nm according to the procedure describe by Armenteros et al. [14]. The inter- and intra -assay coefficients of variation (CVs) were 14% and 8%, respectively. 

#### 2.3.3. Endogenous Antioxidant Enzymes

Analysis of catalase (CAT) and superoxide dismutase (SOD) activities in livers, muscles and blood were carried out by spectrophotometric measurements according to the procedures reported by Carvalho et al. [15]. One unit (U) of catalase was defined as the amount of extract needed to decompose 1 mmol of H_2_O_2_ per min. One unit of SOD was taken as the activity that inhibits the pyrogallol autoxidation by 50%. The inter- and intra -assay coefficients of variation (CVs) were 19% and 11%, respectively.

#### 2.3.4. Quality Parameters in Chicken Breast

Analyses of pH were made in chicken breast muscles at 45 min and 24 h after slaughter by AOAC methods using a CRIMSON pH-meter. Analysis of color in muscles was made by instrumental means (colorimeter MINOLTA) on the surface of chicken breasts. Means of three randomly made measurements on the surface of each breast were obtained. CieLab parameters were used as indicators of redness (a*), yellowness (b*) and lightness (L). Analysis of water holding capacity (WHC) in muscles was made by a centrifugation method as reported by Carvalho et al. [15]. A total of 5 g of ground breast meat was dispensed in test tubes together with 5 stainless steel balls and subjected to 1500 g for 5 min. The water loss upon centrifugation was used for calculating the WHC (g water retained per g of sample and eventually expressed in %). 

#### 2.3.5. Statistical Analysis

Statistical analysis was made using an SPSS program. Data was analyzed for normality (Kolmogorov–Smirnov’s test) and homoscedasticity (Levene’s test). A Student *t*-test was applied in order to find differences between the two diets in regard to all measurements. Quantitative but ordinal variables (incidence of myopathies for instance) were analyzed using a non-parametric test (Kruskal-Wallis). Pearson correlations (r) were also calculated between variables. Significance level was set at *p* < 0.05.

## 3. Results and Discussion

### 3.1. Production Parameters in Broilers as Affected by Dietary Mg Supplementation

Overall, the health status of the birds was normal throughout the assay and no major issues were noticed in this regard. There were 5 casualties (0.63%) in the starting phase (0–14 days), 5 more (0.63%) in the growing phase (14–28 days) and 6 (0.77%) in the finishing phase (28–42 days). This mortality showed no connection with the treatments. The mortality during the 42 days was 16/792 (2.02%). The growth of the broilers was within expected ranges considering the assay conditions (genetic line, diets and type of growing system). The live weight increased from 42.6 g (day 1) to 2.4 kg (day 42) and no significant differences were observed between treatments at any of the stages of production. The main production parameters such as daily weight gain (DWG, g/d); daily average feed consumption (DAFC, g/d) and the conversion index (CI, g/g) were also within expected ranges and no differences were found between groups (Table 2). Hence, supplementation with Mg at 0.3% in the diet (3000 ppm) had no negative impact on production parameters and, furthermore, no evident effects on health/mortality were observed. This is an interesting finding as many previous authors have warned against the supplementation of Mg given the potential negative consequences on Ca absorption and bone formation, catharsis and laxative problems [7,11]. While some interactions with Ca were observed (reported and discussed in following section), no impact on physiological processes was observed and none of the aforementioned problems were found in the present study. Though some authors have reported that 0.3% Mg in the diet may be within the limit of promoting catharsis in broilers, the dose employed (0.3%) of the TIMAB product during the last two stages of production in the present experiment may be appropriate given the lack of disadvantages in terms of animal production parameters. In fact, some relevant positive effects on meat quality were found (described below). It is also known that, as well as for other micronutrients, inorganic Mg (such the commercial Mg product used in this study) is less available than organic Mg (i.e., Mg L-aspartate) [16].

### 3.2. Mineral Composition of Tissues from Broilers as Affected by Dietary Mg Supplementation

Table 3 shows the concentration of Mg^2+^, Ca^2+^ and P in breast, blood and liver of experimental broilers. Supplementation with inorganic Mg had an effect on the levels of Mg^+2^ in blood and liver while the concentration of Mg^+2^ in the muscle was similar between treatments. Stillmak and Sunde [17] postulated blood levels of Mg as the most reliable indicator of Mg absorption and availability. These results agree with that report and confirm that liver may also accurately reflect dietary supplementation with Mg. As expected, interactions were found with Ca^2+^ but not with P. Compared to those fed the basal diet, animals supplemented with Mg had significantly lower levels of Ca^2+^ in blood and breast while no differences were found for the liver. Several mechanisms would explain the observed effects of dietary Mg on Ca metabolism and include, among others, the fact that dietary Mg may hinder intestinal uptake of Ca^2+^. Intestinal absorption of Ca^2+^ and Mg^2+^ occurs by a common mechanism for divalent ions [18]. It has also been proposed that as Mg intake increases, P absorption progressively decreases, possibly because of formation of insoluble Mg–phosphate complexes [19]. This was not observed in the present experiment, as P concentration was similar between treatments in the three tissues under study. While these interactions have been proposed to be responsible for an interference with the process of calcification, this event was not observed in the present study. Taking into account the available literature, this may have happened when Mg is provided at high levels in the first weeks of life. Because of this, the 0.3% supplementation was given to treated broilers in the grower and finisher feeds (from day 14 until the end of the assay). 

### 3.3. Antioxidant Enzymes and Oxidation Markers in Tissues from Broilers as Affected by Dietary Mg Supplementation

Table 4 shows the activities of two major endogenous antioxidant enzymes in broiler tissues, namely, catalase (CAT) and superoxide dismutase (SOD). Data show that Mg promoted the expression and/or activities of the antioxidant enzymes, particularly CAT, in broiler tissues. This more intense CAT activity was reflected in protection against protein oxidation in the plasma and liver. The concentration of MDA in the breast was in general very low and consistent with that reported in literature for raw chicken breast [2]. Mg supplementation had no significant effect on MDA levels. It is known that Mg plays a role in the oxidative status of broilers but the underlying molecular mechanisms are not fully understood. According to Afanasev et al. [18] the antioxidant activity of Mg depends on its radical scavenging properties and on the inhibition of xanthine oxidase and nicotinamide adenine dinucleotide (NADPH) oxidase activities. Consistently, Liu et al. [16] postulated that Mg could inhibit LOX in chicken muscles through the reduction of reactive oxygen species production. Accordingly, Mg deficit is known to cause an increase in hydrogen peroxide production and a depletion of catalase in chick embryo hepatocytes in vitro, subsequently leading to oxidative stress and biological damage [20]. It is worth highlighting that both Mg and catalase are located in the cytoplasm, and this may explain why the antioxidant protection observed on liver proteins (located in the cytoplasm) was not observed on unsaturated lipids (located in biological membranes). Mg supplementation provided the liver and plasma proteins with higher oxidative stability while no effect was observed in the breast muscle. The effect of Mg supplementation on CAT activity deserves a more detailed examination that may lead to elucidate whether the molecular mechanisms involve the implication of this element or any other metabolite in upregulating CAT gene expression, or if Mg may be implicated in activating the antioxidant actions of this enzyme. 

### 3.4. Quality Parameters in Breast Muscles from Broilers as Affected by Dietary Mg Supplementation

Table 5 shows the pH, instrumental color parameters and WHC of chicken breast muscles as affected by the dietary supplementation with Mg. No significant differences were observed for the pH that ranged within expected values for breast muscles from 42-day old broilers. Differences were found for the instrumental color, which is commonly used as the most reliable indicator of freshness and consumer acceptance of poultry color [21]. Muscles from treated broilers had a significantly higher WHC than those from the basal counterparts. WHC largely depends on the functionality and integrity of myofibrillar proteins and guarantees (1) the suitability of such muscle to be subjected to assorted processing technologies (formation of emulsions, gels, etc.) and (2) desirable sensory properties of the meat, as the WHC is directly linked to juiciness and other texture properties of meat [2]. It is worth noting that the effect of Mg on this functional property may be related to their ability to protect proteins against undesirable chemical modifications (including oxidative reactions) [22]. While no differences for protein carbonyls were found between breast samples in the present experiment, the higher WHC in breast muscles from Mg-treated broilers reflects the benefit of such supplementation of protein functionality. It is not ruled out that the effect of Mg supplementation on WHC would have been exerted through antioxidant mechanisms even if the DNPH method did not show significant differences. This method has been largely criticized for not showing accurate results [15,22] and, furthermore, other forms of oxidative damage to proteins with relevant effects on protein functionality (i.e., formation of cross-links) were not measured here. In skeletal muscle, the activity of endogenous antioxidant enzymes (including catalase) has been directly related to protection against protein oxidation and improved protein functionality [23].

### 3.5. Breast Myopathy Incidence in Broilers as Affected by Dietary Mg Supplementation

The carcasses from all the slaughtered animals were assessed for the incidence of WB and WS. Each carcass received a mark in the range from #1 (normal) to #3 (severe) for each myopathy in accordance to the scale described in the Material and Methods section. The frequency results are shown in Table 6. Both myopathies were found in chicken carcasses from all dietary treatments. Taking the results all together, white striping (WS) was more frequent than wooden breast (WB); around 40% of all carcasses (regardless of dietary treatment) had some symptoms of WB while almost 47% of the carcasses had some degree of WS. Since the reported incidences of these myopathies by private companies and/or scientific studies are scarce, it is rather difficult to assess whether these numbers are within an expected range. However, some recent studies [24,25] and the data privately provided by some slaughterhouses and experts in the field agree that the occurrence of both myopathies (particularly WS) is high and the present values may be in a normal range for animals from the same genetic and feeding background. According to a recent review [8], the incidence of WS and WB is so high in European countries that it may be rather difficult to find a single carcass from animals selected for fast growth with no symptom of any of these myopathies. According to the observations made by the experts, there is a clear connection between weight at slaughter, breast size and incidence of both myopathies. Around 95% of the breast in the upper percentile line for weight showed some kind of myopathy. It is also worth mentioning that around 70% of the animals with WB also had WS while the latter also appeared with no apparent symptoms of WB, which is in fact in accordance to the literature [8]. 

The manipulation of the basal diet in the present study significantly reduced the incidence of both myopathies. The incidence of WB decreased from 62% in normal broilers to 31% in broilers supplemented with the TIMAB Mg product. Similarly, supplementation with Mg reduced the incidence of WS from 66% in the control to 38%. At this point, the underlying mechanisms by which the supplementations with Mg reduced the occurrence of myopathies in broiler breasts are not clear. However, two likely hypotheses could be formulated. Since WB and WS are both related to fast growth, and apparently the genetic background (considering always fast-growth genetic lines) plays a minor role in the incidence [8], it seems obvious that other environmental factors, including the feed composition and management could actually play a more critical role in the onset of the myopathies. On this line, this micronutrient could have modulated the development of broiler, and in particular the growth of the breast muscle, to keep it within physiological parameters. As for other myopathies, the onset of both WS and WB seems to be caused by an ischemic condition of the breast muscle: The size of the breast is so marked in the heavy breeds that the muscle becomes strangulated by inelastic fascia and the sternum [8]. The result may be an occlusion of the blood vessels leading to a degeneration of the muscle, with lipidosis, fibrosis and impairment of other physiological functions. While the ischemia and necrosis may not be as evident as for the deep pectoral disease, the symptoms reflect muscle degeneration and a lack of harmony between the growth of muscle and that of the adjoining structures (including connective tissue and bones). It may be possible that Mg, involved in many physiological processes including Ca and vitamin D metabolism in bones, could have improved the concurrence of muscle growth and the supporting bones to avoid the pathological conditions that may lead to the onset of the myopathies. It is also known that a better-balanced condition of Ca, Mg, and P in the ration is essential to normal growth and functioning, and it may be possible that the supplementation of Mg provided in the present study could have led to a more equilibrated muscle/bone growth as recently suggested by Hashtak and Rodehutscord [7]. The other possibility would imply the involvement of the supplemented micronutrients in endogenous antioxidant defenses that may have alleviated the oxidative stress that is known to occur during the onset of these myopathies [9,10]. As aforementioned, Mg was also found to contribute to strengthen the endogenous antioxidant defenses, though this role is much less known than for other microelements such as Se or Cu. 

**Figure 1:**
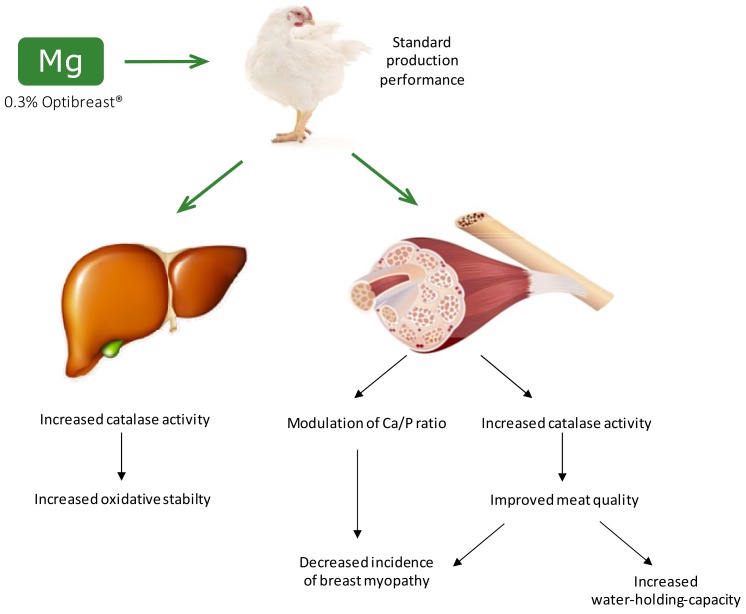
Graphical representation of main benefits of Mg supplementation on broiler redox-status, oxidative stability and meat quality.

Samples identified to have a level of severity of WB and/or WS of 3–5 were analyzed separately for proximate composition, pH, water holding capacity (WHC), color and oxidative stability (Table 7). Likewise, the analyses made on the chicken breast samples as affected by the dietary treatments did not include these abnormal samples so that the effects of dietary supplementation may not be affected by out-of-range values. In line with previous reports [21,25], muscles affected with WS had less water and higher lipid content than normal samples. In addition to significant differences in lightness between samples, it is particularly remarkable the loss of protein functionality in abnormal samples, with WHC being significantly lower than in the normal counterparts. This may be, at least for WB samples, partly explained by the higher susceptibility of these samples to protein oxidation. The oxidative damage to proteins, which may have occurred during the onset of the pathology or as a consequence of it, has been proposed to impair the functional properties of myofibrillar proteins [2]. The higher susceptibility to protein oxidation, in particular of the WB samples, may also be linked to the lower activity of endogenous antioxidant enzymes in these samples. This result expands some knowledge on this relevant topic, as it illustrates the potential connection between this emerging chicken breast abnormality, oxidative stress and specific biochemical measurements (protein oxidation). Figure 1 shows the main benefits of Mg supplementation, in terms of oxidative stability, meat quality and incidence of breast abnormalities. 

## 4. Conclusions

Mg supplementation at 0.3% using Optibreast^®^ has no negative influence on production parameters and had no impact on health issues of any kind. It had a clear interaction with the absorption/accumulation of Ca in blood and breast but this effect had no negative influence on any of the production parameters under study. Mg supplementation had no influence on the extent of lipid oxidation in any of the tissues under study. Conversely, Mg supplementation protected against protein oxidation in liver and plasma of broilers. This effect may be related to the increased activity of catalase in the tissues under study. Mg supplementation had positive effects on particular meat quality parameters such as color and water holding capacity (WHC). The latter may have, in turn, other positive effects on relevant sensory traits such as juiciness. Finally, Mg supplementation reduced the incidence of white striping and wooden breast myopathies to almost half of the occurrence of such defects in animals fed basal Mg levels. 

## Figures and Tables

**Table 1:** Composition of basal diet.

Ingredients (%)	Starter 0–14 Days	Grower 14–28 Days	Finisher 28–42 Days
Corn	30.95	36.98	40.99
Soy flour 44% PB	36.91	31.41	27.74
Wheat	25.00	25.00	25.00
Soy oil	2.71	2.64	2.60
Calcium phosphate	1.58	1.53	1.50
Calcium bicarbonate	1.42	1.17	1.01
Vitamin premix ^1^	0.40	0.40	0.40
DL-Methionine	0.29	0.23	0.19
L-Lysine HCL	0.23	0.18	0.14
Salt	0.22	0.24	0.25
Sodium bicarbonate	0.21	0.19	0.18
L-Threonine	0.07	0.04	0.02
**Calculated analysis ^2^ (%)**			
Metabolizable energy, kcal/kg	2900	2960	3000
Dry matter	88.39	88.23	88.12
Starch	34.83	38.61	41.14
Total protein	22.10	19.94	18.50
Neutral detergent fiber	9.22	9.09	9.01
Ash	6.11	5.55	5.18
Ether extract	4.93	4.97	5.00
Total fiber	3.21	3.07	2.98
Lys total	1.35	1.16	1.04
Lys dig.	1.19	1.02	0.91
Calcium	1.05	0.93	0.85
Met+Cys total	0.97	0.86	0.78
Trh total	0.88	0.77	0.70
Met+Cys dig.	0.87	0.76	0.69
Thr dig.	0.75	0.65	0.59
Total phosphorous	0.74	0.71	0.69
Met total	0.61	0.53	0.47
Met dig.	0.58	0.49	0.43
Available phosphorous	0.45	0.43	0.42
Trp total	0.27	0.24	0.22
Trp dig.	0.23	0.20	0.19
Sodium	0.16	0.16	0.16
Magnesium ^3^	0.11	0.10 (0.38)	0.12 (0.39)
α-tocopherol ^4^	40.3	52.3 (39.8)	51.8 (46.9)

^1^ Composition per kg of feed (free of Mg): vitamin A (E 672): 10,000 IU; vitamin D3 (E 671): 2000 IU; vitamin K3: 2.0 mg; vitamin B1: 2.0 mg; vitamin B2: 5.0 mg; vitamin B6: 3.0 mg; vitamin B12: 12.0 μg; niacin: 40.0 mg; calcium pantothenate: 10.0 mg; folic acid: 1.0 mg; biotin: 0.1 mg; choline chloride: 400 mg; Cu (CuSO_4_·5H_2_O): 8.0 mg; Fe (FeCO_3_): 60.0 mg; I (IK): 2.0 mg; Mn (MnO): 70.0 mg; Se (Na_2_SeO_3_): 0.15 mg; Zn (ZnO): 80.0 mg. ^2^ Based on FEDNA (Spanish Foundation for the Animal Nutrition Development) [13]. ^3^ Concentration of Mg and tocopherol in Mg diet into brackets. ^4^ expressed as mg/kg.

**Table 2:** Effect of the dietary treatment on the daily weight gain (DWG, g/d); daily average feed consumption (DAFC, g/d) and the conversion index (CI, g/g) of the broilers.

Starting Stage	DWG	DAFC	CI
Basal	24.6	38.4	1.57
Mg	23.9	37.6	1.58
SEM ^1^ (*n* = 9)	0.44	0.92	0.05
*p-*value	0.15	0.63	0.28
**Growing Stage**	64.9	103.2	1.59
Basal	64.9	103.2	1.59
Mg	64.7	101.9	1.58
SEM ^1^ (*n* = 9)	0.75	0.89	0.02
*p-*value	0.72	0.30	0.48
**Finishing Stage**			
BASAL	79.8	162.3	2.04
Mg	80.1	159.7	2.00
SEM ^1^ (*n* = 9)	1.26	1.55	0.02
*p-*value	0.85	0.41	0.58

^1^ Standard error of the mean.

**Table 3:** Effect of the dietary treatment on the concentration of Mg^2+^, Ca^2+^ and P (mg/kg) in breast, blood and liver of experimental broilers.

Tissue	Basal	Mg	SEM ^1^	*p-*Value
**Breast**				
Ca^2+^	104.9	81.1	6.18	<0.05
Mg^2+^	251.2	249.8	6.90	0.48
P	256.4	259.0	5.90	0.12
**Blood**				
Ca^2+^	197.9	147.7	13.02	<0.05
Mg^2+^	64.1	79.0	5.17	<0.05
P	98.90	99.21	5.60	0.31
**Liver**				
Ca^2+^	81.1	78.6	5.7	0.36
Mg^2+^	91.4	112.3	6.8	<0.05
P	288.5	299.6	6.5	0.38

^1^ Standard error of the mean. Total observation numbers: 64 (32 per dietary treatment).

**Table 4:** Effect of the dietary treatment on catalase (CAT) and superoxide dismutase (SOD) activities (U/mg), and concentration of thiobarbituric acid reactive substances (TBARS) (mg/kg) and protein carbonyls (nmol/mg protein) concentration in tissues from experimental broilers.

Tissue	Basal	Mg	SEM ^1^	*p-*Value
**Breast**				
CAT	0.51	0.94	0.06	<0.05
SOD	14.53	14.47	0.05	0.640
TBARS	0.05	0.05	0.01	0.621
Protein carbonyls	0.70	0.72	0.02	0.503
**Plasma**				
CAT	1.29	3.70	0.46	<0.05
SOD	19.23	19.23	0.16	0.265
TBARS ^2^	3.64	3.65	0.31	0.656
Protein carbonyls	11.27	9.75	0.16	<0.05
**Liver**				
CAT	6.26	9.20	0.17	<0.05
SOD	16.07	16.17	0.78	0.546
TBARS	0.15	0.15	0.01	0.478
Protein carbonyls	5.06	3.24	0.06	<0.05

^1^ Standard error of the mean, ^2^ Expressed as µmol/mL. Total observation numbers: 64 (32 per dietary treatment).

**Table 5:** Effect of the dietary treatment on general physicochemical parameters and water holding capacity (WHC, %) of breast muscle of experimental broilers.

Parameter	Basal	Mg	SEM ^1^	*p-*Value
pH 45 min	6.26	6.29	0.24	0.65
pH final	5.76	5.76	0.08	0.54
Redness	3.81	4.06	0.99	0.32
Lightness	51.76	53.89	1.67	<0.05
Yellowness	2.18	1.34	0.48	<0.05
WHC	88.67	94.37	2.23	<0.05

^1^ Standard error of the mean. Total observation numbers: 64 (32 per dietary treatment).

**Table 6:** Effect of the dietary treatment on the incidence (number of animals with a given severity assessment) of wooden breast (WB) and white striping (WS) upon slaughter.

Myopathy	Basal	Mg	*p*-Value
WB#1-NORMAL	12	22	<0.05
WB#2-MILD	16	8	<0.05
WB#3-SEVERE	4	2	<0.1
WS#1-NORMAL	11	20	<0.05
WS#2-MILD	17	10	<0.05
WS#3-SEVERE	4	2	<0.1

Severity scale: WB#1: normal; WB#2: slight bulging and toughness; WB#3: severe bulging, toughness and congestion. WS#1: normal; WS#2: thin stripes (<1 mm); WS#3: thick stripes (>1 mm). Total observation numbers: 64 (32 per dietary treatment).

**Table 7:** Modifications of physicochemical properties in breast chicken muscles affected by wooden breast (WB) and white striping (WS) abnormalities.

Parameter	Normal	WB	WS	SEM ^1^	*p-*Value
Moisture ^2^	74.14 ^a^	74.21 ^a^	71.14 ^b^	0.25	<0.05
Lipid ^2^	1.64 ^b^	1.74 ^b^	2.18 ^a^	0.08	<0.05
Protein ^2^	24.19	23.93	23.52	0.31	0.12
pH 45 min	6.28	6.23	6.17	0.08	0.61
pH final	5.73	5.75	5.76	0.02	0.75
Redness	4.09	4.63	4.86	0.33	0.36
Lightness	53.29 ^a^	51.48 ^b^	51.93 ^b^	0.92	<0.05
Yellowness	1.99	1.82	1.97	0.54	0.34
WHC ^2^	93.08 ^a^	88.49 ^b^	87.56 ^b^	0.86	<0.05
MDA ^3^	0.047	0.040	0.057	0.01	0.21
Protein carbonyls ^4^	0.66 ^b^	1.18 ^a^	0.78 ^ab^	0.02	<0.05

^1^ Standard error of the mean; ^2^ water holding capacity, %; ^3^ mg MDA/Kg sample; ^4^ nmol carbonyls/mg protein. Total observation numbers: 64. ^a,b^ Different superscripts denote statistical differences between means.
